# Amplification of the *Insulin-Like Growth Factor 1 Receptor* Gene Is a Rare Event in Adrenocortical Adenocarcinomas: Searching for Potential Mechanisms of Overexpression

**DOI:** 10.1155/2014/936031

**Published:** 2014-07-10

**Authors:** Tamaya Castro Ribeiro, Alexander Augusto Jorge, Madson Q. Almeida, Beatriz Marinho de Paula Mariani, Mirian Yumi Nishi, Berenice Bilharinho Mendonca, Maria Candida Barisson Villares Fragoso, Ana Claudia Latronico

**Affiliations:** ^1^Unidade de Endocrinologia do Desenvolvimento, Laboratório de Hormônios e Genética Molecular LIM42, Brazil; ^2^Unidade de Endocrinologia Genética/LIM25, Brazil; ^3^Unidade de Suprarrenal, Disciplina de Endocrinologia e Metabologia, Hospital das Clínicas da Faculdade de Medicina da Universidade de São Paulo, São Paulo, Brazil

## Abstract

*Context*. *IGF1R* overexpression appears to be a prognostic biomarker of metastatic pediatric adrenocortical tumors. However, the molecular mechanisms that are implicated in its upregulation remain unknown.* Aim*. To investigate the potential mechanisms involved in *IGF1R* overexpression. *Patients and Methods*. We studied 64 adrenocortical tumors. *IGF1R *copy number variation was determined in all patients using MLPA and confirmed using real time PCR. In a subgroup of 32 patients, automatic sequencing was used to identify *IGF1R* allelic variants and the expression of microRNAs involved in *IGF1R* regulation by real time PCR.* Results*. *IGF1R* amplification was detected in an adrenocortical carcinoma that was diagnosed in a 46-year-old woman with Cushing's syndrome and virilization.* IGF1R* overexpression was demonstrated in this case. In addition, gene amplification of other *loci* was identified in this adrenocortical malignant tumor, but no *IGF1R* copy number variation was evidenced in the remaining cases. Automatic sequencing revealed three known polymorphisms but they did not correlate with its expression. Expression of miR-100, miR-145, miR-375, and miR-126 did not correlate with *IGF1R* expression.* Conclusion*. We demonstrated amplification and overexpression of* IGF1R *gene in only one adrenocortical carcinoma, suggesting that these combined events are uncommon. In addition,* IGF1R* polymorphisms and abnormal microRNA expression did not correlate with *IGF1R* upregulation in adrenocortical tumors.

## 1. Introduction

Adrenocortical tumors are rare endocrine malignancies [1]. However, the incidence of these tumors is remarkably high in Southern Brazil, where it is estimated to be 10–15 times greater than the worldwide incidence [2, 3]. These tumors can occur in all age groups and have been characterized as having a bimodal age distribution, with the first peak occurring before 5 years of age and the second between the fourth and fifth decades [3].

The insulin-like growth factor (IGF) signaling system plays an important role in the growth and development of many tissues, including the adrenal gland [4]. Insulin-like growth factors are mitogens that regulate cell proliferation, differentiation, and apoptosis by interacting with the IGF1 receptor (IGF1R) [5]. This system has also been implicated in various pathophysiological conditions and is thought to play a particular role in tumorigenesis [6]. The IGF1R pathway is important in promoting oncogenic transformation, growth, and survival of cancer cells [7], and* IGF1R* overexpression has been demonstrated in many cancers [8, 9].

We previously demonstrated* IGF1R *overexpression in 65% of children and 13% of adults with adrenocortical tumors [10]. Interestingly, this* IGF1R* upregulation was a predictor of metastases in children with adrenocortical tumors. Additionally, a selective IGF1R kinase inhibitor exhibited antitumor effects in adult and pediatric adrenocortical tumor cell lines, suggesting that IGF1R inhibitors represent a promising therapy for human adrenocortical carcinoma [10]. The* IGF1R* upregulation was confirmed in another cohort of pediatric patients [11]. The molecular mechanisms that lead to increased* IGF1R* expression in these tumors remain unexplained. In this study, we investigated whether* IGF1R* gene amplification and allelic variations could be implicated in* IGF1R* upregulation. In addition, the expression of four distinct microRNAs (miRNA) that were involved in* IGF1R* regulation was evaluated.

## 2. Patients 

The study included 64 Brazilian patients with sporadic adrenocortical tumors (Table 1). Written informed consent, as approved by the Ethics Committee of the Hospital of Clinics from the University of São Paulo, São Paulo, SP, Brazil, was obtained from all participants in this study. The consent was acquired directly from the subject if he/she was an adult or from the parent/guardian otherwise. Samples of sporadic adrenocortical tumors were obtained from 25 children and adolescents (18 girls and 7 boys; 1 to 18 years of age) and 39 adult patients (34 women and 5 men; 19 to 73 years of age). The diagnosis of malignancy in the pediatric group was established in 8 of the 25 adrenocortical tumors by advanced tumor stage (III or IV) and/or poor clinical outcome. Adult adrenocortical tumors were classified according to the Weiss criteria and included 23 adrenocortical adenomas (Weiss score < 3) and 16 carcinomas (Weiss score ≥ 3).* IGF1R* expression was previously studied in 45 patients (24 children/adolescents and 21 adults) using real time PCR [10], and the overexpression of this receptor was observed in 17 children and 5 adults [10]. In 32 out of these 45 patients automatic sequencing was used to identify* IGF1R* allelic variants and the expression of microRNAs involved in* IGF1R* regulation was determined by real time PCR. Eight normal adrenal gland cortices, which were obtained from children and adults (range of age: 1 to 72 years) during renal surgery or autopsies, were used as controls. The fragments of adrenal cortices were properly selected by an experienced pathologist.

## 3. Molecular Analysis

### 3.1. DNA Extraction

Genomic DNA was extracted from frozen tumor samples using a Wizard Genomic DNA Purification Kit (Promega, WI, USA) and was stored at −35°C. The concentration and purity of the genomic DNA were measured, using spectrophotometer at an absorbance of 260 and 280 nm.

### 3.2. Multiplex Ligation-Dependent Probe Amplification (MLPA)

The* IGF1R *copy number of the adrenocortical tumors samples was measured using the SALSA MLPA* kit* P217 IGF1R (MRC-Holland, Amsterdam, Netherlands). This molecular assay was designed to detect deletions/duplications of one or more exons of the* IGF1R *(chromosome 15q26) and* IGFBP3* (chromosome 7p13) genes. The P217 IGF1R probe mix contained 22 specific probes for* IGF1R*, 5 probes for* IGFBP3,* and 9 control probes. The* FGFR4* and* NSD1* genes analysis was performed using the SALSA MLPA kit P026-C1 Sotos (MRC-Holland, Amsterdam, The Netherlands), which contains two probes for* FGFR4 *gene and 24 probes for NSD1 gene (5q35.1). MLPA was performed with a total of 200 ng of genomic DNA for each sample, as previously described [12]. The PCR products were submitted to capillary electrophoresison an ABI Prism 310 Genetic Analyzer (PE Applied Biosystems, The Perkin-Elmer Corporation, CA, USA), and the MLPA results were analyzed using the Genescan 3.7 software and further evaluated using the Excel spreadsheet software (Microsoft Corporation, Redmond, WA, USA). The tumor sample normalized peak height was then divided by the average normalized peak height of normal adrenals. Dosage quotient areas outside the range of 0.70–1.3 were considered abnormal. In addition, visual comparison of peak profiles was performed, three control samples were included in each MLPA experiment, and all results were confirmed by two independent tests.

### 3.3. SYBR Green Real Time PCR

SYBR Green real time PCR for the* IGF1R* gene (GenBank accession number NC_000015.9 for genomic and NM_000875.3 for mRNA sequences) was used to confirm the MLPA results for all of the adrenocortical tumors that were studied. The candidate gene copy number was measured using an ABI Prism 7000 instrument (Applied Biosystems, Forster City, CA, EUA). The Niemann-Pick disease type C1 gene (*NPC1*) (GenBank accession number NC_000018.9 for genomic and NM_000271.4 for mRNA sequences) was used as a house-keeping gene, and specific forward and reverse primers for* IGF1R *were used. The PCR was performed in 25 *μ*L containing 2.5 *μ*L of DNA (50 ng), 10 pmol of each primer, and the power SYBR Green PCR master mix (Applied Biosystems, Forster, CA). The thermal cycling conditions consisted of denaturation for 10 min at 95°C, followed by 40 cycles of denaturation for 15 sec at 95°C and annealing/extension for 1 min at 60°C. A cycle threshold (CT) value in the linear range of amplification was selected for each sample in triplicate and normalized to* NPC1*. The* IGF1R *dosage was determined using the 2^−ΔΔCT^ method [13]. The normalized value (ΔCT) for each tumor sample was then compared with the mean ΔCT of five normal adrenals to generate a fold change ratio (normal = 1) that was multiplied by 2 to obtain the copy number (normal = 2).

### 3.4. *IGF1R *Sequence Analysis

*IGF1R *sequence analysis was performed in 32 (19 children-adolescents and 13 adults; 19 adenomas and 13 carcinomas) of 64 patients with adrenocortical tumors. Among these patients, 18 (52.6%) exhibited* IGF1R *overexpression. The entire coding region and exon-intron boundaries of* IGF1R *were amplified from genomic DNA using PCR. The oligonucleotide sequences and PCR conditions are available upon request. The PCR products were pretreated with a combination of shrimp alkaline phosphatase and exonuclease I (United States Biochemical Corp., Cleveland, OH) and directly sequenced using the BigDye TM terminator cycle sequencing ready reaction kit (PE Applied Biosystems, Foster City, CA) in an ABI Prism 310 automatic sequencer.

### 3.5. Expression of MicroRNAs

Some microRNAs can regulate IGF-1R expression. Four microRNAs that were related to* IGF1R* regulation were selected: miR-100, miR-145, miR-375, and miR-126 [11, 14–16]. MicroRNA analysis was performed in 32 samples of adrenocortical tumors and in the same tumors that were submitted to automatic sequencing. To measure microRNA expression, RNA was extracted and quantitative real time PCR was performed. Single-stranded cDNA was synthesized from 1 mg of total RNA using Megaplex RT Primers, Human Pool A v2.1 (PN 4399966, Applied Biosystems), and the TaqMan MicroRNA Reverse Transcription Kit (PN 4366596, Applied Biosystems). Twenty-five nanograms of cDNA was used as a template in a 20-*μ*L PCR reaction, and the PCR products were amplified using specific primers (TaqMan MicroRNA Assays: hsa-mir100-ID: 002142, hsa-mir 145-ID: 002278, has-mir375-ID: 000564, has-mir126-ID: 002228, Applied Biosystems) and the Taqman Universal Master Mix II, with no UNG (PN 4440040, Applied Biosystems). The PCR products were detected using the ABI Prism 7000 Sequence Detection System (Applied Biosystems). RNU 44 (ID: 001094, Applied Biosystems) and RNU 48 (ID: 001006, Applied Biosystems) were used as house-keeping genes. The relative expression levels were analyzed using the 2^−ΔΔCT^ method. A commercial pool of normal human adrenals RNA was used as a reference sample (Clontech, Palo Alto, CA).

### 3.6. P53 Analysis

Genomic DNA was extracted from frozen tumor specimens using a QIAmp DNA mini Kit (QIAGEN, Hilden, Germany). The entire exon 10 was amplified by PCR using the following intronic primers: 5′-GCTGTATAGGTACTTGAAGTGCAG-3′ and 5′-GATGAGAATGGAATCCTATG-3′. The amplification protocol consisted of denaturing at 94°C for 5 min, followed by 35 cycles consisting of annealing at 50°C for 30 sec, primer extension at 72°C for 30 sec, and denaturing at 94°C for 30 sec. All amplified fragments were examined on 1% agarose gel electrophoresis. The PCR products were pretreated with an enzymatic combination of exonuclease I and shrimp alkaline phosphatase (United Stated Biochemical Corp., Cleveland, OH) and directly sequenced using the BigDye terminator cycle sequencing ready reaction kit (PE Applied Biosystems, Foster City, CA) in an ABI PRISM 310 automatic sequencer (Perkin-Elmer Corp.).

## 4. Results

### 4.1. *IGF1R* Copy Number

**Figure 1:**
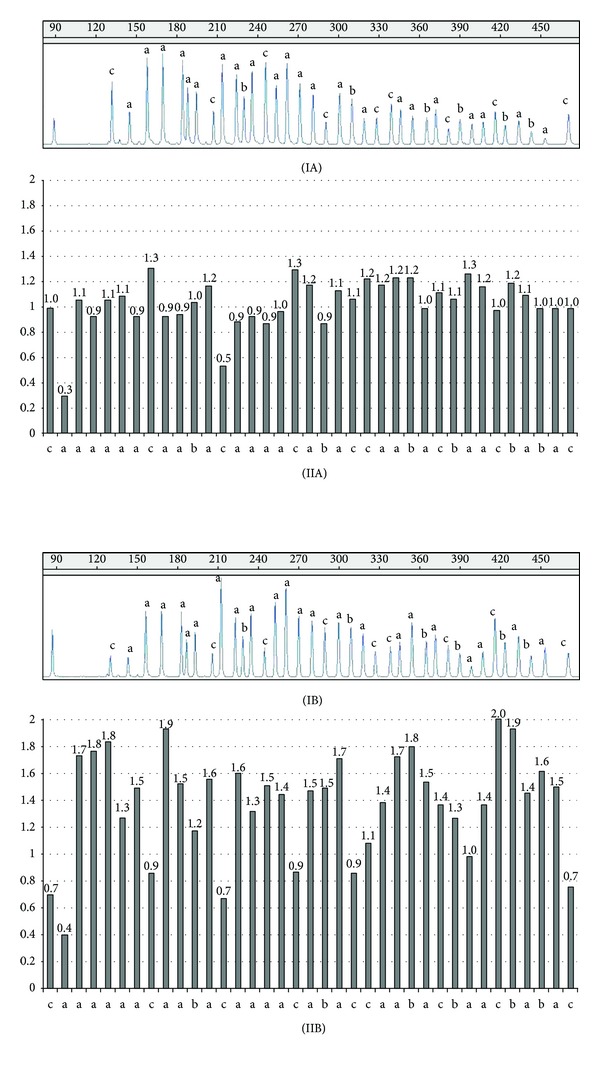
(I) Representative MLPA peak patterns of the* IGF1R* and* IGFBP3* genes. The numbers in the top portion of panels (IA) and (IB) represent the fragment size in the capillary electrophoresis analysis. (II) Histogram showing the normalized data with results of three control individuals and internal controls (reference probes). Normalized relative values, ranging within a confidence interval of 0.7 to 1.3, were established by control data variations and correspond to two gene copies in the genotype. The values in *y*-axis correspond to the normalized relative value and the number on the top of each bar represents the normalized value for each probe. (A) Normal adrenal gland. (B) Adrenocortical tumor with* IGF1R* and* IGFBP3* amplification. Some reference probes in this tumor are also amplified. (a): twenty-two* IGF1R* probes. (b): six* IGFBP3* probes. (c): reference probes.

*IGF1R* amplification was detected using MLPA and confirmed using real time PCR in an adrenocortical carcinoma that was diagnosed in a 46-year-old female patient with Cushing's syndrome associated with clinical virilization (Figure 1). The tumor's size was 10 × 8 × 6 cm, and it was classified as ENSAT stage III and presented a Weiss score of 7 upon histopathological analysis.* IGF1R* mRNA levels were 5 times higher in this tumor than in the normal total adrenal gland pool. Additionally, the amplification of the* IGFBP3, FGFR4 *(data not shown), and* NSD1 *(data not shown) genes was also detected in this carcinoma, but* IGF1R* amplification was not demonstrated in the remaining cases with adrenocortical tumors.

### 4.2. *IGF1R* Sequence Analysis

Three distinct previously described exomic polymorphisms were detected in* IGF1R: *exon 11 rs_3743262 (10.5%, genotype frequency), exon 16 rs_2229765 (65.8%, genotype frequency), and exon 21 rs_17847203 (7.9%, genotype frequency). No correlation between the* IGF1R* variants and their expression was demonstrated in the adrenocortical tumors. In addition, another 6 previously known polymorphisms were identified in the intronic regions (rs_7174918, rs_2272037, rs_951715, rs_1464430, rs_4486868, and rs_2593053).

### 4.3. MicroRNA Expression

**Figure 2:**
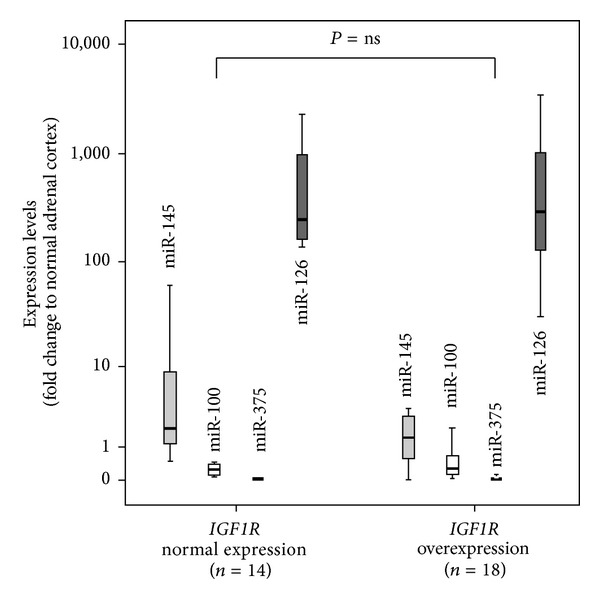
Expression of microRNAs 145, 100, 375, and 126 in adrenocortical tumor samples with normal and increased* IGF1R* expression. No difference of microRNA expression was observed for all microRNAs. (miR-145 *P* = 0.19, miR-100 *P* = 0.61, miR-375 *P* = 0.49, and miR-126 *P* = 0.81).

Real time PCR analysis revealed that miR-100 (median 0.3 (from 0.07 to 1.97)) and 375 (median 0.05 (from 0.01 to 0.27)) were downregulated in adrenocortical tumors. This reduction was observed in benign and malignant tumors and was not associated with increased expression of* IGF1R*. miR-145 (median 2.2 (from 0.01 to 12.6)) had variable expression in the adrenocortical tumors that were evaluated; however, these values were not correlated with the expression values of* IGF1R* in adrenocortical adenomas and carcinomas. miR-126 (median 596.6 (from 30.8 to 2667.4)) was overexpressed in benign and malignant adrenocortical tumors and was not related to* IGF1R* expression (Figure 2).

### 4.4. Statistical Analysis

Statistical analysis was performed using SPSS Software (PASW version 19.0; SPSS Inc., Chicago, IL). Continuous data are expressed as median values (from minimum to maximum). Differences in expression levels between two groups were analyzed by means of the two-tailed Mann-Whitney *U* test.

## 5. Discussion

IGF1R has potent mitogenic, antiapoptotic, and transforming activities [17]. Normal growth and differentiation are result of a fine balance between the process of cell proliferation and death, and the disruption of one or more factors involved in these actions can result in pathologic phenotypes, including malignancy [17]. Indeed,* IGF1R* is overexpressed in several primary tumors and cancer-derived cells [18, 19]. It has been demonstrated that IGF1R expression is a fundamental prerequisite for cellular transformation because enhanced IGF1R levels and IGF1 signaling are considered key factors for the cell to adopt the proliferative and oncogenic pathways [20]. Increased expression of the human IGF1 receptor promotes ligand-dependent neoplastic transformation in different cells [6, 21]. On the other hand, the absence or decrease of levels of the IGF1 receptor prevents malignant growth and transformation [22].

We identified* IGF1R *overexpression in metastatic pediatric adrenocortical tumors and found that it was a prognostic biomarker of advanced tumors in these children [10]. In present study, we hypothesized that* IGF1R *overexpression in adrenocortical tumors could be caused by gene amplification, allelic variants, and dysregulated microRNA expression. In fact,* IGF1R* amplification has been reported in malignant melanomas [23], breast cancers [24], pancreatic adenocarcinomas [25], gastric cell lines [26], rhabdomyosarcomas [27], Wilms' tumors [28], and gastrointestinal stromal tumors [29]. Moreover,* IGF1R *amplification was associated with cellular transformation and tumor progression [30].

The* IGF1R* copy number in 64 adrenal tumor samples was analyzed using MLPA in this study. Amplification of* IGF1R* was detected in only one adrenocortical carcinoma (Weiss score 7) that was diagnosed in a woman who had an endocrine hyperfunction that was characterized by hypercortisolism and hyperandrogenism. This patient was treated with mitotane and she had a favorable evolution over the previous 10 years. As expected, the adrenocortical tumor with* IGF1R* amplification diagnosed in this patient had higher* IGF1R* mRNA levels compared to normal adrenal samples. MLPA analysis also demonstrated a multiplegene amplification, suggesting a potential aneuploidy.* IGF1R* gene amplification was not demonstrated in the remaining adrenocortical tumors, which suggested that other mechanisms could be implicated in* IGF1R* upregulation. Additionally only the previously known exomic and intronic polymorphisms were observed in tumors with or without* IGF1R* overexpression.

Stimulatory and inhibitory transcription factors determine the level of* IGF1R *expression, and, consequently, the proliferative cell status. The* IGF1R* gene is controlled by a number of tumor suppressors, including p53, which is the most frequently mutated molecule in human cancer. P53 was able to suppress* IGF1R* promoter activity by approximately 90%, whereas its mutant forms were associated with* IGF1R* upregulation [31]. A specific germinative mutation (p.R337H) that affected the tetramerization domain of p53 has been identified in high frequency in Brazilian children with adrenocortical tumors [32, 33]. This unique p53 defect was detected in 22 of the 64 (34.3%) patients of the Brazilian cohort that was studied here (data not shown). Nevertheless,* IGF1R *overexpression and gene amplification were not associated with the presence of this p53 mutation. In addition, the adrenocortical carcinoma with* IGF1R* amplification did not harbor the p.R337H mutation in blood or tumor tissue DNA (data not shown).

MicroRNA abnormalities are potential mechanisms that may be associated with* IGF1R* overexpression. Calin et al. [34] demonstrated that more than 50% of microRNA genes were located in cancer-associated genomic regions. In the present work, we demonstrated that the expression of miR-100 and 375 was decreased in benign and malignant adrenocortical tumors; however, no correlation was found between the selected microRNAs and* IGF1R* expression. miR-145 also did not correlate with* IGF1R* expression values. Our data corroborate with those from previous studies that showed that miR-100 and 375 expression was decreased in adrenocortical tumors and could be tumor suppressor microRNAs [11, 35]. In addition, Doghman et al. [11] showed that miR-100 regulated the expression of* IGF1R* and mTOR signaling in adrenocortical cells by modulating the expression of key proteins involved in these pathways [11]. miR-375 is also downregulated in gastric cancers and hepatocellular carcinomas [15]. Previous studies in esophagus carcinoma cells showed that miR-375 has a tumor suppressor effect by inhibiting* IGF1R* [15]. Shi et al. [14] identified that IGF1R and its docking protein, the insulin receptor substrate-I, are targets of miR-145. miR-126 was overexpressed in benign and malignant adrenocortical tumors. This microRNA suppresses endothelial migration by targeting a set of genes in cancer cells that drive endothelial migration, by activating IGF1R, which is a promoter of endothelial migration, and inhibiting the endothelial MERTK receptor, which is a suppressor of endothelial migration [16].

In conclusion, we detected concurrent* IGF1R* gene copy number variation and* IGF1R* overexpression in a single functioning malignant adrenocortical tumor. This data suggests that the* IGF1R* overexpression, which is common in pediatric adrenocortical tumors, is not driven by increased gene copy number. In addition,* IGF1R* polymorphisms and the abnormal expression of the microRNAs miR-100, miR-145, miR-375, and miR-126 did not correlate with* IGF1R* overexpression in these tumors.

## Supplementary Material

Primers that were used in the *IGF1R * sequence analysis and the PCR conditions. Clinical and molecular characteristics of 64 patients with adrenocortical tumors, including *IGF1R * expression and p53 data.

## Figures and Tables

**Table 1:** Clinical characteristics of 64 patients with adrenocortical tumors.

	Children		Adults	
	Clinically benign *n* = 18	Clinically malignant *n* = 7	Adenomas *n* = 24	Carcinomas *n* = 15
Sex (F : M)	3.5 : 1	1.3 : 1	11 : 1	4 : 1
Clinical presentation (*n*)				
Cushing	2	0	20	3
Virilizing	12	4	0	1
Mixed	4	2	2	7
Nonfunctioning	0	0	2	4
Feminizing	0	1	0	0

F: female; M: male.
