# Supplementary material for: Amplification of the Insulin-Like Growth Factor 1 Receptor Gene Is a Rare Event in Adrenocortical Adenocarcinomas: Searching for Potential Mechanisms of Overexpression
Source: Biomed Res Int. 2014 Jul 10;2014:936031. doi: 10.1155/2014/936031 (PMC4119736; doi:10.1155/2014/936031)
Supplement: Supplementary file 1 — Primers that were used in the IGF1R sequence analysis and the PCR conditions. Clinical and molecular characteristics of 64 patients with adrenocortical tumors, including IGF1R expression and p53 data. [file 936031.f1.docx]

**Supplementary Material**

**Table 1**- Primers used in the *IGF1R* sequence analysis

| **Exon** | **Primer** | **Annealing** |
| --- | --- | --- |
| 1 | 5’-GGG AGC GAA GAC TGA GTT TG- 3’ | 57 ºC |
|  | 5’- GCT GTC AGG ACG GAG AAA GA- 3’ |  |
| 2A | 5’-GAG TGT GTT GCA CAG CGT CT-3’ | 57 ºC |
|  | 5’ -GAT GAC CAG GGC GTA GTT GT-3’ |  |
| 2B | 5’ -ACC TCC ACA TCC TGC TCA TC- 3’ | 58 ºC |
|  | 5’ -GCA AGT GTC TAG CCC AGG AG- 3’ |  |
| 3 | 5’ -GTG CTG GTG GTA GCA GTG AA- 3’ | 55 ºC |
|  | 5’ -ATC GAA ACC AGC AAG CAA GT- 3’ |  |
| 4 | 5’ -TTG GGG GTG AGA TAC CAT GT- 3’ | 54 ºC |
|  | 5’ - ACG TGC CAA TGG CTA AAA GA- 3’ |  |
| 5 | 5’ -CTG CCG TTG AAT TGT TCT CA- 3’ | 55 ºC |
|  | 5’ -AGG GAG GGC TCT TAC CTC AG- 3’ |  |
| 6 | 5’ -TGA ATC ATG TTG CTG CCA TT- 3’ | 56 ºC |
|  | 5’ -GAG GAA AGC TGC CAT CAC AC- 3’ |  |
| 7 | 5’ -ACG AGA AAG CCA CTG AGG AA- 3’ | 55 ºC |
|  | 5’ -TCT GCT GAC CCC CTG TAT TC- 3’ |  |
| 8 | 5’ -GAA GCC CAG AGT CCA GTC AC- 3’ | 55 ºC |
|  | 5’ -GCC AGA AAA TGG CAC AGA AG- 3’ |  |
| 9 | 5’ -TTT CAA AAT AAG GTT CAC TTT TCA C- 3’ | 56 ºC |
|  | 5’ -CCA GAA GAT AGC TGG TTT CCA- 3’ |  |
| 10 | 5’ -CTC CCA AAG TGC TGG GAT TA- 3’ | 55 ºC |
|  | 5’ -CCC AGC TGC TGA CTT ACA CA- 3’ |  |
| 11 | 5’ -GGG GCT CAA TAG CTC CTT CT- 3’ | 57 ºC |
|  | 5’ -CTG GTC TAC ACG CCA CCT G- 3’ |  |
| 12 | 5’ -GTG GCT GAT CTC CAC TGT CC- 3’ | 53 ºC |
|  | 5’ -AAG ACC CAA ATC CTC ACC AA- 3’ |  |
| 13 | 5’ -GTC CCC AGT GTG GTG AGT TT- 3’ | 57 ºC |
|  | 5’ -GCA CCT TAG CAT GAC CCT TC-3’ |  |
| 14 | 5’ -TGT ATG ATG GGG AGT AAA CGA A- 3’ | 59 ºC |
|  | 5’ -TCC TGG AAG AAC ATT TGA TGG- 3’ |  |
| 15 | 5’ -GCT CTC CCC ATT CTG TTC TG- 3’ | 55 ºC |
|  | 5’ -AAC CTC CTT CCT TTC CCA AA- 3’ |  |
| 16 | 5’ -GGC TTA GAG TTC CCC CAA AG- 3’ | 57 ºC |
|  | 5’ -CAG GGG TCT GAA GGT AGC AG- 3’ |  |
| 17 | 5’ -TGC TAC CTT CAG ACC CCT GT- 3’ | 55 ºC |
|  | 5’ -GAC ACA GCA TTT CCT TGC AG- 3’ |  |
| 18 | 5’ -GGT GCC CAG ATT GAA CAA AG- 3’ | 55 ºC |
|  | 5’ -TCT CCA GGG GCA GAC TAA TG- 3’ |  |
| 19 | 5’ -CGT GTC TGT GTC TTG CCT TG- 3’ | 55 ºC |
|  | 5’ -TGT GGC TCA CAC AAC ACT GA- 3’ |  |
| 20 | 5’ -GCT CGG GAT GTA AGA AGT GC- 3’ | 55 ºC |
|  | 5’ -TCC TTC CAC AAG GAC AAA CC- 3’ |  |
| 21 | 5’ -AGA AAG CCA GGG ATG GAG AG- 3’ | 56 ºC |
|  | 5’ -CAG GAG GCT TGT GAA TGG AT-3’ |  |

PCRs were all performed in the same thermal cycler (Veriti® 96-Well Thermal Cycler, Applied Biosystems, Foster City, CA, USA) followed by 40 cycles. Annealing period was 30 seconds for all set of primers.

**Table 2** - Clinical and molecular characteristics of 64 patients with adrenocortical tumors.

| **Patient** | **Age**  **(Years)** | **Sex** | **Clinical**  **Presentation** | **Diagnosis** | **Weiss** | **Mac- Farlane** | **p53 (p.R337H)** | **mRNA**  ***IGF-1R*** |
| --- | --- | --- | --- | --- | --- | --- | --- | --- |
| 1 | 1 | F | C/V | Adenoma | 3 | II | present | 1,69 |
| 2 | 73 | M | C | Adenoma | 0 | I | absent | NA |
| 3 | 29 | M | C | Carcinoma | 8 | IV | absent | 1,06 |
| 4 | 2,1 | F | V | Adenoma | 1 | I | present | 5,27 |
| 5 | 36 | F | C | Adenoma | 1 | II | present | NA |
| 6 | 17 | F | C | Adenoma | 2 | I | absent | 1,21 |
| 7 | 2,2 | M | V | Adenoma | 4 | II | present | 1,63 |
| 8 | 52 | F | C | Adenoma | 2 | I | absent | NA |
| 9 | 70 | F | C | Adenoma | 0 | I | absent | NA |
| 10 | 2,1 | F | V | Adenoma | 1 | I | present | 1,11 |
| 11 | 2,1 | M | V | Adenoma | 5 | II | present | 2,28 |
| 12 | 27 | F | C | Adenoma | 1 | I | absent | 0,61 |
| 13 | 29 | F | C/V | Carcinoma | 4 | II | absent | 1,69 |
| 14 | 52 | F | NF | carcinoma | NA | IV | absent | NA |
| 15 | 17 | M | F | Carcinoma | 7 | II | absent | 1,32 |
| 16 | 46 | F | C | Adenoma | 0 | II | absent | NA |
| 17 | 22 | F | C | Adenoma | 2 | I | absent | NA |
| 18 | 1,1 | M | C/V | Adenoma | 1 | I | present | 1,16 |
| 19 | 12 | F | V | Adenoma | 2 | II | absent | NA |
| 20 | 15 | F | V | Carcinoma | 6 | IV | present | 9,95 |
| 21 | 33 | M | NF | Carcinoma | 5 | IV | present | 0,46 |
| 22 | 36 | F | C/V | Adenoma | 3 | I | absent | 1,43 |
| 23 | 30 | M | NF | Carcinoma | 4 | III | absent | 0,46 |
| 24 | 3 | M | V | Carcinoma | 7 | III | present | 10,62 |
| 25 | 37 | F | C | Adenoma | 2 | I | absent | 11,48 |
| 26 | 35 | F | C | Adenoma | 1 | I | absent | 0,47 |
| 27 | 19 | F | C | Carcinoma | 6 | 2 | absent | NA |
| 28 | 49 | F | C | Adenoma | 2 | 1 | absent | 0,65 |
| 29 | 47 | F | C | Adenoma | 1 | I | absent | NA |
| 30 | 46 | F | C/V | Carcinoma | 7 | III | absent | 5 |
| 31 | 23 | F | C/V | Carcinoma | 5 | III | absent | NA |
| 32 | 17 | F | C/V | Carcinoma | 7 | IV | present | NA |
| 33 | 34 | M | C | Adenoma | 1 | I | absent | NA |
| 34 | 2,5 | F | V | Adenoma | 7 | II | present | 4,67 |
| 35 | 1,6 | F | V | Adenoma | NA | I | present | 3,34 |
| 36 | 1 | F | C/V | Carcinoma | 4 | II | present | 4,04 |
| 37 | 19 | F | C/V | Carcinoma | 8 | II | present | 3,43 |
| 38 | 23 | F | C/V | Carcinoma | 6 | IV | present | 0,9 |
| 39 | 41 | F | C/V | Adenoma | 0 | II | absent | NA |
| 40 | 1,2 | F | V | Adenoma | 5 | I | present | 2,62 |
| 41 | 64 | F | C | Adenoma | 0 | I | absent | 2,67 |
| 42 | 31 | F | C | Adenoma | 2 | I | absent | NA |
| 43 | 27 | F | C | Adenoma | 1 | I | absent | 1,89 |
| 44 | 28 | F | C | Adenoma | 0 | 1 | absent | 0,45 |
| 45 | 39 | F | C | Adenoma | 1 | I | absent | 0,77 |
| 46 | 26 | F | C/V | Carcinoma | 8 | II | present | NA |
| 47 | 66 | F | NF | Adenoma | 4 | II | absent | 2,83 |
| 48 | 9 | M | C | Adenoma | 2 | I | absent | 1,47 |
| 49 | 1,1 | F | V | Adenoma | 2 | I | absent | 3,51 |
| 50 | 2 | F | C/V | Adenoma | 7 | II | absent | 9,5 |
| 51 | 37 | F | C | Carcinoma | 3 | II | absent | NA |
| 52 | 2,6 | M | V | Carcinoma | 5 | III | present | 7,4 |
| 53 | 45 | F | NF | Adenoma | 0 | I | absent | 0,98 |
| 54 | 17 | F | V | Carcinoma | 4 | II | absent | 2,95 |
| 55 | 2,8 | F | V | Adenoma | 2 | I | present | 2,1 |
| 56 | 2,2 | F | V | Adenoma | 2 | II | present | 2,39 |
| 57 | 37 | F | C/V | Carcinoma | 6 | II | absent | NA |
| 58 | 24 | F | C | Adenoma | 1 | I | absent | 1,89 |
| 59 | 22 | F | V | Carcinoma | 7 | III | absent | 0,83 |
| 60 | 39 | F | C | Adenoma | 2 | I | absent | NA |
| 61 | 41 | F | C | Adenoma | 1 | I | absent | 1,22 |
| 62 | 44 | F | NF | Carcinoma | 8 | IV | absent | 2,62 |
| 63 | 2,5 | F | V | Adenoma | 1 | I | present | 2,94 |
| 64 | 9 | F | C/V | Adenoma | 7 | II | absent | 13 |

C/V: Mixed; C: Cushing; V: Virilizing; NF: Nonfunctioning; F: Feminizing, F:Female, M: Male: NA: Data not available
